# Substituent Effects on Crystal Engineering of DNBT-Based Energetic Cocrystals: Insights from Multiscale Computational Analysis

**DOI:** 10.3390/ma18132995

**Published:** 2025-06-24

**Authors:** Lu Shi, Min Liu, Shangrui Xie, Song Li, Shuxin Liu, Shen Yuan, Xiaohui Duan, Hongzhen Li

**Affiliations:** 1 College of Chemistry and Materials Engineering, Mianyang Teachers’ College, Mianyang 621000, China; m19981747764@163.com (M.L.); xieshangrui1219@163.com (S.X.); lisong197411@163.com (S.L.); liushuxin88@126.com (S.L.); 2 Department of Intelligent Manufacture, Automation Research Institute Co., Ltd. of China South Industries Group Corporation, Mianyang 621000, China; myyuan89@126.com; 3 Key Laboratory of Environment-Friendly Energy Materials, Southwest University of Science and Technology, Mianyang 621010, China; duanxiaohui@swust.edu.cn; 4 Institute of Chemical Materials, China Academy of Engineering Physics (CAEP), Mianyang 621900, China; hongzhenli@caep.cn

**Keywords:** energetic cocrystals, DNBT, intermolecular interactions, detonation performance

## Abstract

The substituent effects on crystal stacking topology and stability of the 5,5-dinitro-2H,2H-3,3-bi-1,2,4-triazole (DNBT) and its three energetic cocrystals with 1,3,5-trinitrobenzene (TNB), 2,4,6-trinitrotoluene (TNT), and picric acid (PA) were systematically investigated through combined density functional theory (DFT) calculations and classical molecular dynamics (MD) simulations. The interaction mechanism and detonation performance of the three energetic cocrystals were implemented to the electrostatic potential (ESP), Hirshfeld surface analysis, radial distribution function (RDF), binding energy, and detonation parameters. In contrast to N-H⋯O interactions in DNBT, three cocrystals exhibited more distinctly weak C-H⋯O intermolecular hydrogen bonds and NO_2_-π stacking interactions to stabilize the lattice. Notably, the highest binding energy of PA/DNBT shows the largest stability and lowest impact sensitivity is related to the more intermolecular interactions. Although the introduction of substituents slightly affects the crystal density of DNBT crystals, it significantly reduces the impact sensitivity. Moreover, the balanced detonation performance and impact sensitivity of DNBT-based cocrystals make it a candidate to expand the applications of DNBT crystals. These findings contribute to a broadened understanding of construction and design strategies for the energy release mechanisms of energetic compounds with the azoles ring family.

## 1. Introduction

High-energy density materials (HEDMs) that consist of nitro compounds are important for their military and civil application [1,2,3,4,5]. Notably, many efforts have been devoted to nitrogen-rich compound points for their safety, low toxicity, high energy release efficiency, and outstanding energetic properties in industrial processes [6,7,8,9]. Due to the significant advantages of the azoles ring family (pyrazoles, triazoles, and tetrazoles), which possesses high nitrogen content and low hydrocarbon content resulting in their combustion product being an eco-friendly low-molecular-weight gas N_2_; this can reduce the environmental and toxicological hazards of the propellants currently used [10,11]. Furthermore, the conjugated system of high energy C-N, N-N (160 kJ·mol^−1^) and N = N (418 kJ·mol^−1^) bonds formed by large π-bonds in the five-membered ring makes triazoles excellent foundation frameworks for a new explosive design, with a high enthalpy of formation, high thermal stability, and low sensitivity to impact, friction, and discharge [12,13].

As one of the azole explosives, 5,5-Dinitro-2H,2H-3,3-bi-1,2,4-triazole (DNBT) and its analogues possess high detonation velocities and lower impact sensitivity compared to HMX, making them suitable for applications as a prospective high-energy density material in the field of insensitive munitions [14,15,16]. Energetic cocrystals have been widely considered a typical application, especially for designing balanced sensitivity and detonation performance EMs [16,17,18,19,20,21]. Due to the high nitrogen content within the aromatic rings, and unlike traditional nitroesters, the nitramine energetics of DNBT are of particular interest from a crystal engineering perspective, owing to their advantage as good hydrogen bond donors (primary and secondary amines).

For example, three DNBT cocrystals of 2:1 ANTA/DNBT cocrystal, 1:1 DNPP/DNBT cocrystal and 2:1 3,4-DNP/DNBT cocrystal were developed in 2015, which possess high crystal density and high detonation properties compared to its pure components (detonation velocities > 8000 m/s and pressure > 28 GPa) [22]. The differences in intermolecular forces lead to the formation ratios of different cocrystals, with the same hydrogen bonding motif in their structures and the unique packing. The azole-based energetic molecule DNBT as an ideal choice is successfully cocrystallized with AN and AND to yield the cocrystals 2AN:DNBT and 2ADN:DNBT to replace AP in propellant formulations, which maintain high oxygen balance, and to solve the problem of AN stability and burning in 2024 from Adam J. Matzger’ group [23]. Further, these cocrystals showcase the importance of crystal engineering in energetic cocrystals and the potential for further developing reliable interactions forming energetic cocrystals.

By combining appropriate nitroaromatic components, the cocrystal design strategy can adjust the detonation properties and sensitivity of DNBT crystals and increase the controllability and application range of high-energy density materials. A series of three 1:1 cocrystals with DNBT and 2,4,6-trinitrobenzene (TNT), analogues 1,3,5-trinitrobenzene (TNB), and picric acid (PA) were first discovered by Adam J. Matzger’ group in the review article [24]. The experiment and crystal structure data were recorded in the supporting information. Although the nitrobenzene components combine with DNBT in cocrystals, the crystal densities are different with 1.829, 1.870, and 1.768 g/cm^3^, which ultimately affect the overall performance of the material due to the dependency of the detonation velocity on the effective density of energy in the crystal (higher density leads to higher detonation velocity) [21]. However, the lack of systematic understanding of substituent effects on cocrystal stabilization mechanisms hinders the rational design of azole-based HEDMs. It is worth exploring and discussing where the azoles ring family has a similar performance in structure and properties with a feasible and worthy-of-tracking and follow-up consideration to analyze the structural characteristics and detonation properties of these nitro-compounds from the perspective of the crystal structure and electronic energy state.

Herein, the substituent effects on the crystal structure, intermolecular interactions, and detonation performance of three DNBT-based energetic cocrystals with different nitroaromatic compounds were compared through a systematic and comprehensive theoretical calculation. The molecular structure relies on an electrostatic potential surface and was characterized via an electrostatic potential (ESP) and localized orbital locator-π isosurfaces (LOL-π) analysis. The intermolecular interactions on the crystal stacking topology can also be represented by a Hirshfeld surface, IGMH analysis, and radial distribution function (RDF). Further, binding energies and detonation parameters were computationally predicted to evaluate the energy level of cocrystal explosives, and the corresponding relationship between the crystal characteristic structure, packing mode and detonation performance was established. These results reveal at the molecular level the influence of structural differences on the crystal energy and detonation performance. It is expected to provide a comprehensive understanding of the intermolecular interactions in energetic crystals and help us to understand and apply low-sensitive high-energy compounds.

## 2. Methods

The initial structures of unit cell models for a DNBT crystal and three cocrystals TNB/DNBT, TNT/DNBT, and PA/DNBT were constructed at the Cambridge Crystallographic Data Centre (CCDC numbers are 2070498 for TNB/DNBT, 2070499 for TNB/DNBT and 2070500 for PA/DNBT). A Perdew–Burke–Ernzerhof (PBE) functional within the generalized gradient approximation (GGA), ref. [25] combined with the empirical dispersion correction (DFT-D3), was adopted using the DMOL^3^ (Materials Studio 2017) code based on the exchange–correlation functional DFT method, which is widely used in the geometric optimization and interaction calculations of crystal energetic materials, and showed satisfactory results [26,27]. Geometry optimizations were performed according to a Quasi–Newton algorithm with a BFGS minimizer for both lattice parameters and atomic coordinates. The calculations were performed with all electrons and used a double numerical plus polarization (DNP) atomic orbital basis set [28], and continued with “fine” grid sizes (corresponding to an energy convergence less than 5 × 10^−7^ Hartree) or a density convergence being 10^−6^ during the SCF minimization. The Tkatchenko–Scheffler method was chosen as the basic tuning parameter with the Norm-conserving pseudopotentials Koelling–Harmon relativistic treatment. The energy cutoff was 1080 eV. The total energy of the system ensures convergence under plane wave cutoff energy and k-point grid setting.

Three DNBT-based cocrystals were respectively expanded to a matrix of 6a × 5b × 4c for a DNBT crystal (total 5742 atoms), 4a × 4b × 3c for TNB/DNBT (total 6912 atoms), 4a × 4b × 3c for TNT/DNBT (total 7488 atoms), and 4a × 4b × 3c for PA/DNBT (total 8616 atoms) cocrystals to obtain the comparably sized periodic supercells. Further, the geometry and potential energy of supercells were minimized using the smart method, and then further optimized by the conjugate gradient method to ensure that the system energy was the most stable. Condensed phase optimized molecular potentials for an atomistic simulation studies (COMPASSII) force field was selected due to its validated accuracy for the ammonium nitrate energetic materials containing H, O, and N atoms [29]. Then, molecular dynamics simulations using an NVT ensemble were performed at 298 K and 0 GPa for 200 ps to relax the cell parameters and atom coordinates. A Nose–Hoover thermostat was used for controlling the system temperature. The summation methods for electrostatic and van der Waals were Ewald and atom-based, respectively. The interactions were determined within the cutoff distance of 15.5 Å to deal with the van der Waals long-range interaction more accurately. Next, dynamic trajectories were obtained under three-dimensional periodic boundary conditions in the NVE ensemble via the Parrinello–Rahman approach in conjunction with velocity-Verlet integration with 0.1 fs time-steps (given that the holonomic-constraint algorithm was applied for proton vibrations). The information on atomic trajectories and molecular types is in the whole simulation for 200 ps, output with intervals at every 5000 steps.

Electrostatic potential analysis was realized via calculations of the electron density and population analysis. Hirshfeld surfaces [30,31] to produce two-dimensional fingerprint plots and populations of close interatomic contacts of neighboring molecules in crystals to further explore intermolecular interactions and crystal packing characteristics using the CrystalExplorer 17.5 code [32] LOL-π isosurface and IGMH analysis were conducted by the Multiwfn code based on the wave function obtained by B3LYP/6–31G** basis set [33,34,35].

## 3. Results and Discussion

### 3.1. Crystal Structure Characteristics

**Figure 1:**
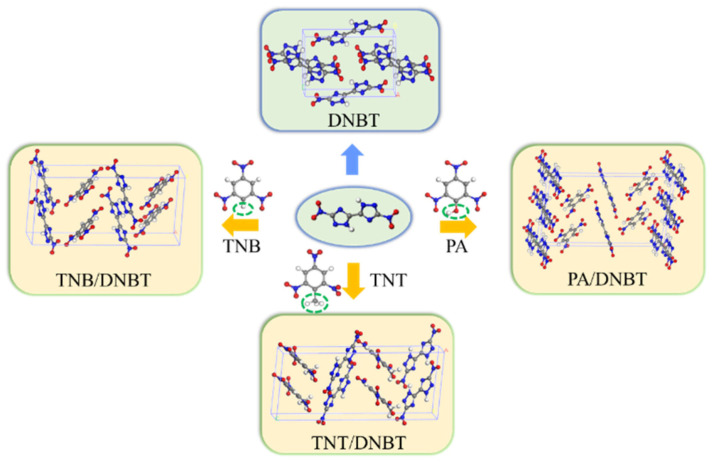
Crystal and molecular structures of DNBT, TNB/DNBT, TNT/DNBT, and PA/DNBT cocrystals.

The molecular composition and crystal structures of DNBT crystals and their cocrystal are shown in Figure 1 after adequate relaxation. DNBT molecules are combined with TNB, TNT, or PA molecules in a stoichiometric ratio of 1:1 to form cocrystals, in which the aromatic derivates act as conformers. Structural distinctions arise from the TNT molecule containing a methyl group (-CH_3_) attached to a conjugated aromatic ring; the PA molecule contains a hydroxyl group (-OH), and TNB is a C-H group. The OH groups can be easily used as hydrogen bond donors to form electron-deficient π systems because of their strong electron absorption induction effect, while the electron cloud density increases due to the methyl group in TNT as an electron donor group (shown in Figure 1). These substituent-induced electronic effects directly govern intermolecular interaction patterns.

**Figure 2:**
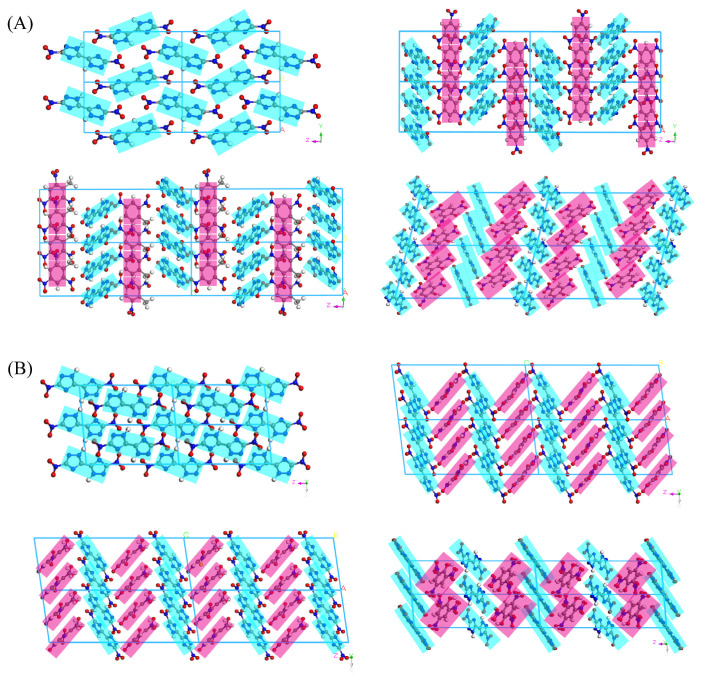
The stacking modes of DNBT, TNB/DNBT, TNT/DNBT, and PA/DNBT cocrystals (**A**,**B**) along the *X*-axis and *Y*-axis directions.

Figure 2 shows the crystal stacking modes of DNBT crystals and their cocrystals along the X and Y axes. The orientations of DNBT molecules in single crystals are in a cross-shaped layered architecture, which relies on forming the hydrogen bond (HB) interactions between the hydrogen atoms attached to the triazole rings and the nitrogen atoms within the triazole rings/the nitro groups on the conformers. For DNBT-based cocrystals, the wavelike-shaped stacking arrangement modes are formed by tapes of the repeat unit that propagate throughout the crystal structures by the nitro groups interacting with the π systems. The PA/DNBT cocrystal exhibits the shortest intermolecular π-π stacking distance (3.12 Å) among the layered, compared to 3.42 Å for TNB/DNBT and 3.35 Å for TNT/DNBT, which correlates with their significantly reduced impact sensitivity relative to DNBT. The DNBT and nitroaromatic molecules both sit on the inversion center and act as hydrogen donors and acceptors. More complex stacking results from the mixed spatial modes composed of multiple spatial orientations of molecules, which give rise to greater resistance to lattice slippage and loading external mechanical stimuli, and further easily dissociate [36].

### 3.2. Electrostatic Potential and π-Electron Analysis

**Figure 3:**
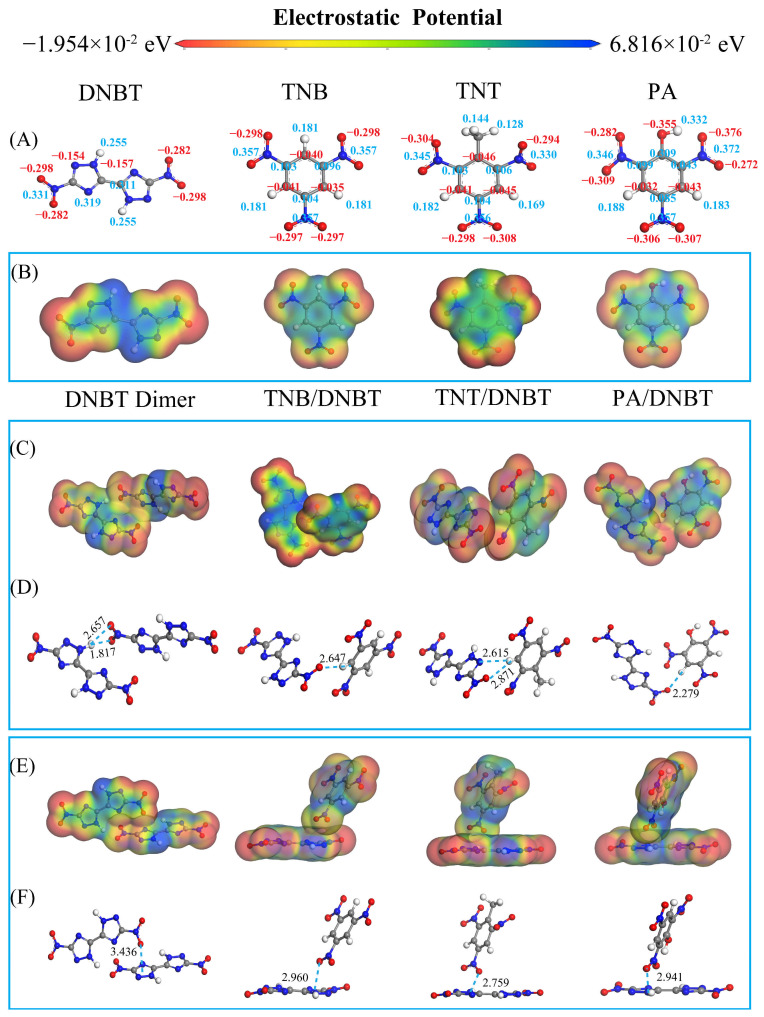
(**A**) Mulliken charge distribution of DNBT, TNB, TNT, and PA molecules. (**B**) electrostatic potential surfaces of explosive small molecules, and (**C**,**D**) typical ESP surfaces of molecular pairs in DNBT-based energetic cocrystals and corresponding typical intermolecular hydrogen bond interactions. (**E**,**F**) ESP surfaces of DNBT with π-conjugated structures and their corresponding intermolecular stacking topologies.

The supramolecular binding motifs between DNBT and nitroaromatic compounds are a matter of great concern. Visual mapping of these interactions through advanced computational tools provides critical mechanistic insights. As an effective means to gain more insights into the prominent physicochemical stabilities of DNBT-based energetic cocrystals, ESP analysis [37,38] was employed. Mulliken charge distribution of DNBT, TNB, TNT, and PA molecules was performed as illustrated in Figure 3A. O atoms of the nitro group exhibit a negative charge (in red) and H atoms exhibit a positive charge (in red). Especially, the π-cloud density in the benzene ring is most increased due to the CH_3_ groups as electron-donating groups in TNT, whereas the OH groups as electron-withdrawing groups reduce the π-cloud density in the benzene ring. Generally, the more negative the electrostatic potential is, the greater the electron density is, the higher the molecular stability is, and the lower the sensitivity is. This may result in a relatively lower impact sensitivity and higher stability in the TNT/DNBT cocrystal.

High positive ESP regions are related to the N-H region on the triazole ring in DNBT and C-H groups in nitrobenzenes, and negative ESP is related to the O atoms of NO_2_ groups, which can easily form the hydrogen bond (HB) N-H⋯O. In addition to the O atom, the N atom on the triazole ring also has electronegativity and can be used as a hydrogen bond acceptor. Typical molecular pairs representing intermolecular interactions were extracted from the optimized cocrystal structures to clarify the driving force of forming energetic cocrystals. As shown in Figure 3B, a weak HB of C-H⋯O was formed between a strong polar O atom in DNBT and C-H groups in neighboring nitrobenzenes molecules in the DNBT cocrystals, with bond lengths > 2.279 Å. In addition, C-H⋯N hydrogen bonds were formed between the CH groups in TNT and act as HB donors and N atoms on the triazole ring in DNBT as acceptors in the TNT/DNBT cocrystal (as shown in Figure 3C). Although there is an OH group acting as a hydrogen bond donor, due to the lack of spatial orientation, there is not an O-H⋯O interaction formed in the PA/DNBT cocrystal. Generally, the weak HB C-H⋯O was observed in energetic cocrystals of nitramine explosives such as HMX and CL-20. However, N-H⋯O and C-H⋯N hydrogen bonds emerge as between explosive conformers as a driving force to stabilize the lattice due to there being more substituents in the nitroaromatic molecules.

**Figure 4:**
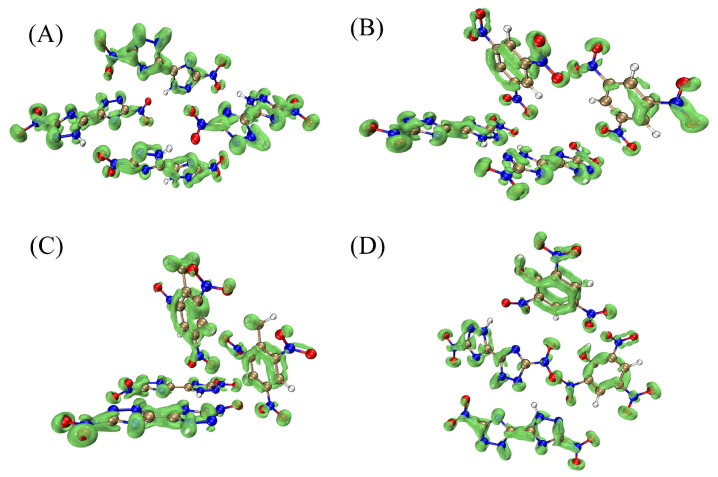
The LOL-π isosurface of the representative clusters in the four crystals: DNBT (**A**), TNB/DNBT (**B**), TNT/DNBT (**C**), and PA/DNBT (**D**) cocrystals.

Further to obtaining more rich interactions in cocrystals, LOL-π isosurfaces serve as a visualization for the degree of π electron delocalization, reflecting the number of occupied π orbitals. The number and distribution of π electrons are directly related to aromaticity and conjugation for a system. Here, Figure 4 shows the LOL-π isosurface of the representative clusters in DNBT, TNB/DNBT, TNT/DNBT, and PA/DNBT cocrystals. The delocalization population of π electrons is observed in the triazole ring and a little at NO_2_ groups in DNBT. The largest π isosurface in the TNT/DNBT cocrystal is attributed to NO_2_-π interactions in the conjugated aromatic ring and delocalized π electrons in the NO_2_ groups, which mainly promote the TNT/DNBT lattice arrangement. This strong π-π crystal stacking can disperse external stimuli through a slip. Therefore, the impact sensitivity of the TNT/DNBT cocrystal should be the lowest among the four crystals, which are also confirmed by the calculation results of *h*_50_ listed in Section 3.5. Figure 3D,E also shows the intermolecular stacking modes where the NO_2_-π (=3.436 Å) are popular in the DNBT crystal, as well as stackings involving lone pair electrons of O atoms in NO_2_ groups of DNBT and the delocalized π rings of the conformers, such as TNB, TNT, and PA. The shortest distance of 2.759 Å between DNBT and TNT molecules is also in agreement with the largest LOL-π isosurface in the TNT/DNBT cocrystal.

### 3.3. Intermolecular Interactions

**Figure 5:**
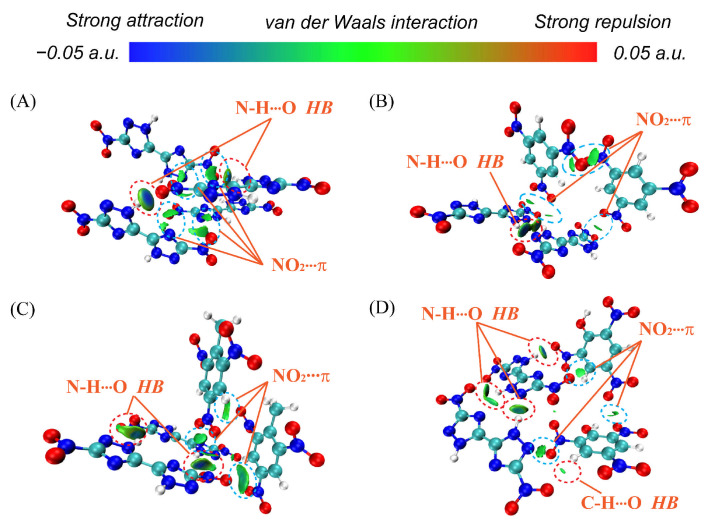
IGMH δg^inter^ = 0.005 a.u. isosurfaces for representing clusters of four crystals: DNBT (**A**), TNB/DNBT (**B**), TNT/DNBT (**C**), and PA/DNBT (**D**) cocrystals. Red and blue circles highlight hydrogen bonds and van der Waals interaction regions, respectively.

Further, we employ the popular IGMH analysis to study the interaction between DNBT and nitroaromatic compounds. The representative clusters of four crystals containing four molecules were chosen for computational costs. Nonhydrogen atoms in triazole and aromatic rings were fixed to the crystal coordinates. The IGMH δg^inter^ = 0.005 a.u. isosurfaces for representing clusters of four crystals are given in Figure 5.

The prominent intermolecular interactions can be viewed, including the four N-H⋯O types of H-bonds between the DNBT molecules. In DNBT-based cocrystals, the sequence of the number of N-H⋯O hydrogen bond sites is according to PA/DNBT > TNT/DNBT > TNB/DNBT. Furthermore, NO_2_-π stacking can be observed in the NO_2_ groups and nitroaromatic ring or triazole ring in DNBT and nitroaromatic compounds. In particular, the green isosurface region is the largest in the TNT/DNBT cocrystal in Figure 5C. Although the NO_2_⋯π interaction is weak, the cumulative effect can also be the driving force for stabilizing the cocrystals. It is also illustrated by the largest binding energy for a TNT/DNBT cocrystal. Furthermore, the weak C-H⋯O HB interactions in the PA/DNBT cocrystal was viewed in OH groups and in PA molecules and do not act as acceptors in HB bonds, similar to CH_3_ groups in TNT/DNBT.

**Figure 6:**
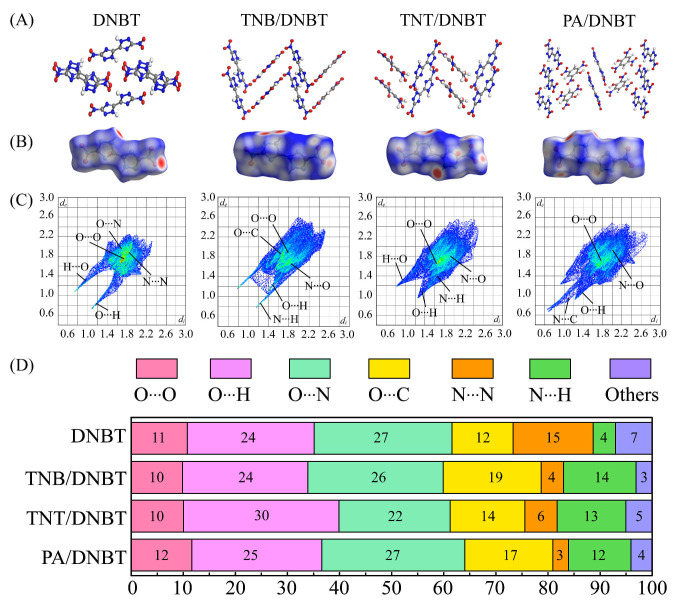
The molecular stacking structures (**A**), Hirshfeld surfaces (**B**), 2D fingerprint plots (**C**) of the DNBT molecules consolidated in various crystals and related populations (%) (**D**) of various close contacts.

As a quantitative complementary means of IGMH analysis, the Hirshfeld surface was analyzed with the position and quantitative distribution of intermolecular interactions from the crystal [39,40,41]. The red dots and blue patches refer to the stronger (intermolecular HB) and weaker (intermolecular vdW) interactions, respectively. Figure 6 illustrates Hirshfeld surfaces and 2D fingerprint plots of DNBT crystal and three DNBT-based cocrystals combined with benzene derivatives of (TNB, TNT, and PA). For four crystals, the contacts are dominated by O⋯H, O⋯O, O⋯N, O⋯C, N⋯N, N⋯H interactions. The four Hirshfeld surfaces appear in a block shape with different flatness, and the red dots thereon are mostly and remarkably positioned on the block sides, representing evident intermolecular HB. This corresponds to the planar molecular structure of DNBT and is consistent with the previous conclusion that Hirshfeld surfaces are relatively flat when impacting insensitive explosives [32]. For the DNBT crystal, an obvious red dot appears in the N-H regions in the triazole ring, which comes from the N-H⋯N and N-H⋯O hydrogen bond as the main stabilizing force for the DNBT crystal. When the nitroaromatic compounds were introduced, the density of close contacts progressively increased according to the sequence of TNB/DNBT < TNT/DNBT < PA/DNBT (Figure 6C). This result is proven in the analysis of IGMH in Figure 5.

Notably, based on the characteristics of the Hirshfeld surface of different cocrystal systems, two-dimensional fingerprints can be used to visualize the characteristics of interactions in different DNBT-based energetic cocrystals. Typically, intermolecular HB and π-π interactions appear as a pair of long spikes (1.2 < di and de < 1.4 Å) and a pair of wings on the bottom left and top right of the two-dimensional fingerprint, respectively. The two-dimensional fingerprints in the four crystals are shown in Figure 6C. It is clear that N-H⋯O is the main HB type in the four DNBT-based cocrystals, and π-π interaction is also evident in the DNBT crystals and TNT/DNBT cocrystals. In the wing-shaped area of each 2D fingerprint plot, the bright part represents the dense contact of O⋯O, O⋯N, O⋯C, and N⋯N interactions at the center of the 2D fingerprint. The corresponding dense contact proportion is located above the Hirshfeld surface markedly in red dots, which comes from the π-π stacking modes of the triazole ring.

As shown in Figure 6D, population analysis can be used to analyze the close contacts in the four crystals further quantitatively. Approximately 50% of the populations of O⋯H and O⋯N close contacts suggest that HB interactions or planar layered stacking play a leading role in DNBT-based cocrystals. The largest proportion of O⋯H contacts was in the TNT/DNBT cocrystal compared with the other three crystals, which is the result of the synergistic stabilization of two types of HB C-H⋯O and C-H⋯N interactions. In addition, the 15% population of N⋯N contacts in DNBT decreases to 4%, 6%, and 3% significantly in three DNBT-based cocrystals reflecting that the wavelike-shaped arrangement modes in DNBT-based cocrystals when the nitroaromatic conformers are inserted, rather than the layered structure in the DNBT crystal. It is worth noting that the population analysis of N⋯H contacts increased from 4% to 14%, 13%, and 12% in turn for TNB/DNBT, TNT/DNBT to PA/DNBT cocrystals. It further demonstrates that C-H⋯N interactions are formed easily between negative N atoms in the triazole ring and H atoms in small aromatic molecules. In general, the populations of O⋯O contacts have a higher proportion of energetic crystals [42]. Here, the nearly population of O⋯O close contacts for DNBT crystal and three DNBT-based cocrystals may have contributed to the abundant electrostatic interaction between adjacent NO_2_ groups. There was no significant difference in intermolecular O⋯C interactions, which was slightly lower in the DNBT crystal, possibly because of the lower content of C and O atoms in DNBT molecules.

**Figure 7:**
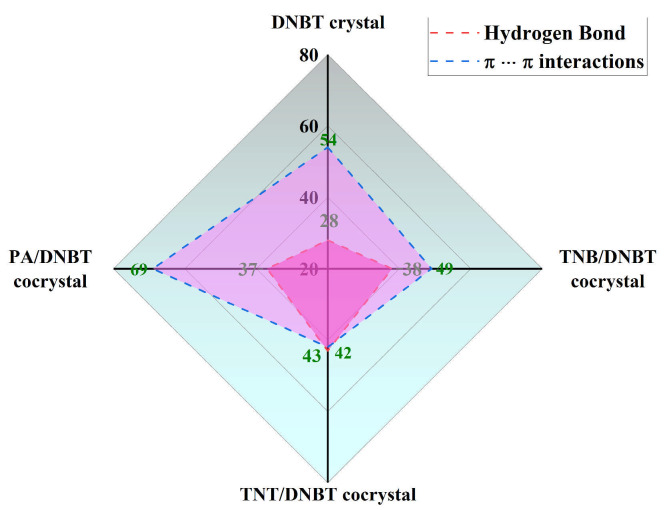
The qualitative contributions (%) from HB and NO_2_⋯π interactions of DNBT crystal and three DNBT-based cocrystals.

Further, based on the populations (%) of various close contacts of Hirshfeld surfaces, the contributions (%) from HB and π-π interactions in DNBT crystal and three DNBT-based cocrystals of TNB/DNBT, TNT/DNBT and PA/DNBT are qualitatively shown in Figure 7. Compared to the interactions of DNBT, the contributions of N-H⋯O and C-H⋯N hydrogen bonds in the three cocrystals are increased. Meanwhile, the largest 69% contributions of π-π interactions are in the PA/DNBT cocrystal, which may be the result of the OH groups as electron-withdrawing groups reduce the π-cloud density in the benzene ring. This also provides powerful support for Section 3.4 that PA/DNBT has a relatively high binding energy.

Even though these four compounds exhibit major hydrogen bonding (HB) and π-π stacking, their surfaces and fingerprint plots differ in shape and distribution of close contact, implying different intermolecular stacking patterns. The other local structures may lead to various types of close contacts of molecular stacking modes. Molecular interaction is the basis for determining crystal topology. However, due to the lack of strong donors or acceptors for intermolecular interaction, such as strong hydrogen bond acceptors and strong hydrogen bond donors, the intermolecular interaction in DNBT-based energetic cocrystals is generally weak. Although the strength is weak, the intermolecular interaction in DNBT-based cocrystals is dominated by electrostatic interactions.

**Figure 8:**
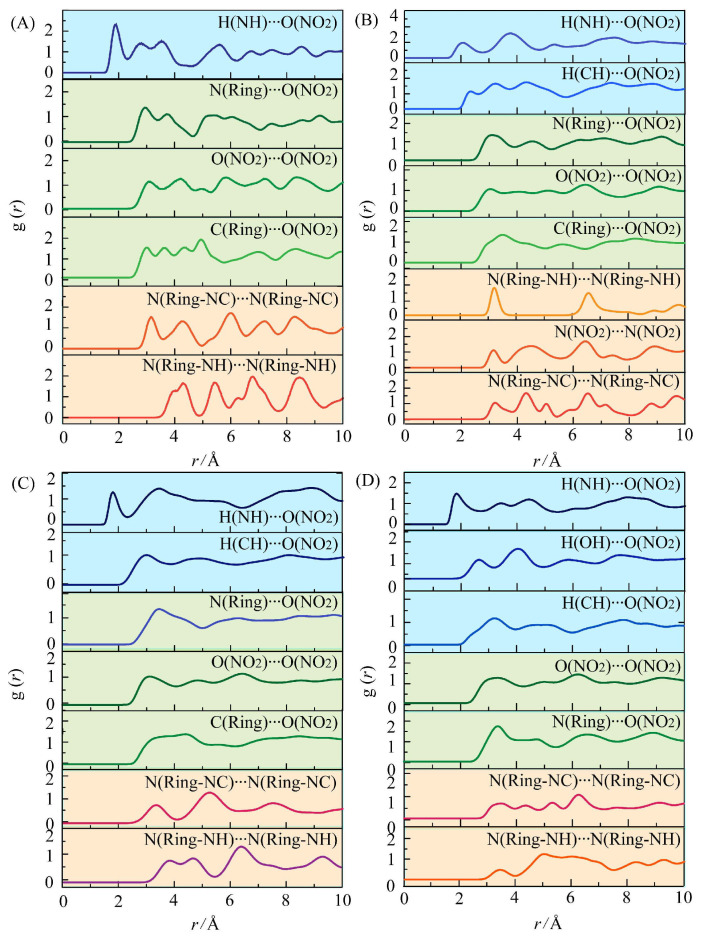
Radial distribution function of DNBT crystal (**A**) and three DNBT-based cocrystals of TNB/DNBT (**B**), TNT/DNBT (**C**) and PA/DNBT (**D**).

Finally, RDFs can be analyzed by measuring the local spatial ordering, which gives a monitor of the probability density g (r) of finding an atom distance from a reference atom. Generally, the interaction distance range (r) for the hydrogen bond is 2.0–3.1 Å, and for strong van der Waals, it is 3.1–5.0 Å. The vdW interactions become negligible beyond 5.0 Å [15]. As shown in Figure 8, the N(ring), H(NH) atom derived from DNBT, H (CH) atom derived from TNT or TNB, H (OH) atom derived from PA. N (NO_2_), O (NO_2_) atom derived from NO_2_ groups. For cocrystals, the A⋯B atom pairs represent the intermolecular interaction between DNBT and nitroaromatic molecules.

In Figure 8A, the first strong peak of g (r) H-O in DNBT crystals appears at 1.9 Å indicating N-H⋯O hydrogen bond between O atoms in NO_2_ groups and NH of the triazole ring of adjacent DNBT molecules. The g (r) N-O at 2.9 Å, g (r) O-O at 2.9 Å, and g (r) C-O at 3.0 Å belong to the strong van der Waals interactions range due to the lack of hydrogen bond donors. The g (r) N-N at 3.2 Å and 4.0 Å between the triazole ring present the strong van der Waals between DNBT molecules with “face to face” π-π stacking modes shown in Figure 3E. These results are consistent with the large proportion of 24% O⋯H and 27% O⋯N interactions in the Hirshfeld surface analysis of DNBT crystals.

For DNBT-based cocrystals, the prominent g (r) H-N below 3.0 Å belong to N-H⋯O interactions, where NH groups act as hydrogen bond donors in DNBT, and O of NO_2_ groups as acceptors (blue shade in Figure 8B–D). Furthermore, g (r) N-O, g (r) O-O, g (r) C-O below 4.0 Å indicate the intermolecular strong van der Waals interactions between DNBT and nitroaromatic compounds. Moreover, the g (r) N-N mainly originated from the triazole ring in adjacent DNBT molecules, reflecting the π-π stacking of the triazole ring. The position of the first strong peaks of g (r) for DNBT-based cocrystals increases according to the sequence of hydrogen bond < van der Waals interactions < π-π stacking shown as blue, green, and orange regions in Figure 8. The formations of DNBT-based cocrystals mainly rely on the H⋯O hydrogen bond interactions between the hydrogen atoms attached to the heterocyclic rings and the nitrogen atoms within the heterocyclic rings/the nitro groups on the conformers.

### 3.4. Binding Energy

The binding energy (*E*_b_) between DNBT and nitroaromatic components of cocrystals was calculated based on the following equation:

(1) $$E_{b}=-E_{inter}=-[E_{total}-(E_{A}+E_{B})]$$

where E_total_ is the total energy of cocrystals A–B, and E_A_ and E_B_ denote the energy of components A (DNBT) and B (TNB, TNT, or PA), respectively. The greater the binding energy is, the stronger is the stability. Table 1 lists the binding energy of three DNBT-based energetic cocrystals, in which the binding energy has the order PA/DNBT > TNT/DNBT > TNB/DNBT. This result is in agreement with the sequence of the HB contact distance obtained by Hirshfeld surface analysis (TNB/DNBT < TNT/DNBT < PA/DNBT) in Figure 6B, which shows that more HB interactions are the main driving force in DNBT-based cocrystals. Generally, the greater the binding energy of energetic crystalline materials is, the greater is the crystal density, which indicates the fairly strong N-H⋯O HB and NO_2_-π interactions between nitroaromatic components and DNBT molecules. Further, electron-withdrawing groups (-OH) in PA/DNBT cocrystals strengthen π electron delocalization and result in higher thermal stability (seen from Figure 3D).

### 3.5. Detonation Performance

Detonation velocity (D) and pressure (P) are the two most important indexes in the detonation characteristics of explosives. Given the qualitative observations of the sensitivity and detonation performance of DNBT-based cocrystals, it was natural to seek theoretical estimates of the heat of explosion for these energetic compounds. According to empirical Formula (2), the explosion pressure (P, GPa) can be obtained [43]:

(2) $$\begin{array}{l} P(\mathrm{GPa})=(-22.3207+104.0393\rho^{2}-10.9781a-1.9967b+5.5619c\\ +5.5392d-23.6834n_{-NH_{X}}-154.0862n_{1}^{0})/10.1972 \end{array}$$

where the coefficients a, b, c, and d represent the number of C, H, N, and O in the crystal formula, respectively. n-NH_x_ represents the number of -NH_2_ and NH^4+^ in an energetic compound. $n_{1}^{0}$ is 1 when *d* > 3 (*a* + *b*), and 0 otherwise. Density at 298 K was predicted from the relations Formula (3) by Politzer [44,45]:

(3) $$\rho =\alpha \frac{M}{V_{m}}+\beta (\nu \sigma_{tot}^{2})+\gamma$$

where the parameters fitted to experimental data are *α* = 0.9183, *β* = 0.0028, *γ* = 0.0443. According to the relation (4) between the detonation pressure and detonation velocity, the detonation velocity (D, km/s) can be obtained, and the adiabatic index (Γ) can be obtained from Formula (5), and the impact sensitivity was predicted from Formula (6) [46]:

(4) $$D={\left(P\times (\Gamma +1)/\rho \right)}^{\frac{1}{2}}$$

(5) Γ=1.819−0.196/ρ+0.712ρ

(6) $$h_{50}=x\sigma_{+}^{2}+y\nu +z$$

The calculated values of the crystal density, detonation velocity, detonation pressure and packing coefficient of the four crystals are listed in Table 2. The detonation velocity (D) and detonation pressure (P) of the DNBT crystal, respectively 8.40 km/s and 32.86 GPa, are in agreement with the results of 8413 m/s and 320 kbar reports in Ref [14] and show the rationality and accuracy of the calculation method of detonation parameters. The detonation velocity (D) and detonation pressure (P) of [44,45] the DNBT-based cocrystal are slightly lower compared to the single crystal, which indicates that the introduction of nitroaromatic compounds will reduce the density of the crystal, and the detonation velocity and detonation pressure of the cocrystals are also lower than that of the DNBT crystal. In addition, the D and P of DNBT-based cocrystals increase with the order of PA/DNBT > TNB/DNBT > TNT/DNBT, which agrees with the lower packing coefficients at 0.68 of TNB/DNBT and TNT/DNBT due to the spatial steric obstruction compared to PA/DNBT at 0.74.

Moreover, the detonation parameters of common high-energy explosives TNT RDX HMX and CL-20 also are listed in Table 2. The detonation velocities of TNB/DNBT, TNT/DNBT and PA/DNBT are 8.17, 7.89 and 8.33 km/s, respectively, and the detonation pressures are 29.52, 27.30 and 30.98 GPa, respectively. It basically reaches the energy equivalent to that of RDX. Furthermore, TNT/DNBT has a slightly lower crystal density due to the lower density of TNT (1.78 g/cm^3^), resulting in a poorer detonation performance. However, its impact sensitivity (*h*_50_) is lower compared to DNBT and comparable to that of RDX, which is much lower than that of HMX and CL-20 [12]. Hence, it is suitable to be developed from DNBT-based cocrystals as a new material in terms of performance and relative safety.

However, the impact sensitivity of cocrystal (*h*_50_ > 40 cm) is lower than that of DNBT crystal (*h*_50_ = 26.38 cm), and the impact sensitivity of TNT/DNBT cocrystal is the lowest (*h*_50_ = 45.3 cm), resulting from the -CH_3_ groups in TNT/DNBT as electron-donating groups further enhancing π-cloud density. Comprehensively analyzing the detonation performance and impact sensitivity of DNBT-based cocrystals, it can be seen that they retain the high detonation velocity and high detonation pressure of the crystal, and also greatly improve the stability. Therefore, DNBT-based cocrystals can be used as an excellent substitute for DNBT single crystals, and further expand the application of rich nitrogen energy material.

## 4. Conclusions

The substituent effects on the formation mechanism of the DNBT crystal and three DNBT-based energetic cocrystals TNB/DNBT, TNT/DNBT, and PA/DNBT were investigated using a DFT calculation and classical molecular dynamic simulations. This work delved into the characteristics of crystal structures by electronic structure and intermolecular interactions analysis, with the overarching aim of elucidating the influences of methyl, hydroxyl, and hydrogen atoms on the formation mechanism of DNBT-based cocrystals. In contrast to triazole explosive DNBT, all three cocrystals exhibited distinct intermolecular interaction motifs in their structures. While the planar stacking was adopted in DNBT, the DNBT-based cocrystals demonstrate a wave-like arrangement, primarily driven by N-H⋯O intermolecular hydrogen bonds and NO_2_-π stacking interactions, as well as weak C-H⋯O HB interactions in PA/DNBT. Moreover, the more stable and lower-impact sensitivity of PA/DNBT cocrystals are presented by the stronger binding energy compared to TNB/DNBT and TNT/DNBT. Although the D and P of DNBT-based cocrystals increase with the order of PA/DNBT > TNB/DNBT > TNT/DNBT, the balanced detonation performance and impact sensitivity of DNBT-based cocrystals can act as excellent candidates as a substitute for the DNBT single crystal. This study’s findings offer a theoretical foundation for advancing our comprehension of the intricate formation mechanisms governing nitrogen-rich salts of energetic compounds, particularly those adorned with various functional groups. The application scope of nitrogen-rich heterocyclic energetic materials is further expanded with its greatly improved sensitivity.

## Figures and Tables

**Table 1:** Binding energy of three DNBT-based energetic cocrystals.

Cocrystals	*E*_A_/Ha	*E_B_*/Ha	*E_Total_*/Ha	*E*_Binding_/kJ mol^−1^
TNB/DNBT	−3567.13	−3380.98	−6948.15	113.38
TNT/DNBT	−3567.10	−3538.05	−7105.19	118.65
PA/DNBT	−3567.13	−3681.75	−7248.93	120.31

**Table 2:** Crystal density (ρ), detonation velocity (D) and detonation pressure (P), packing coefficient, and impact sensitivity of DNBT crystal and its cocrystals.

Crystals	*ρ* (g/cm^3^)		Packing Coefficient	*D* (km/s)		*P* (GPa)		*h*_50_ (cm)
	*DFT*	Ref. [23]		*DFT*	Ref. [38]	*DFT*	Ref. [12]	
DNBT	1.83	1.89	0.78	8.40	8.41	32.86		26.38
TNB/DNBT	1.82	1.829	0.68	8.17		29.52		44.30
TNT/DNBT	1.78	1.768	0.68	7.89		27.30		45.30
PA/DNBT	1.84	1.870	0.74	8.33		30.98		42.05
TNT ^a^		1.78			6.80		19.00	>170
RDX ^a^		1.82			8.75		34.00	46
HMX ^a^		1.91			9.10		39.00	36
CL-20 ^a^		2.04			9.40		42.00	24

^a.^ Data from Ref. [12].

## Data Availability

The original contributions presented in this study are included in the article. Further inquiries can be directed to the corresponding author.
